# A multidimensional platform to support intestinal epithelial and immune cell co-culture

**DOI:** 10.3389/fnut.2026.1769338

**Published:** 2026-06-24

**Authors:** Jennifer L. Walker, Melissa Sprachman, Michaela Welch, Alicia Meehan-Qiu, Kimberly Kelly, Erin Shaughnessey, Heather Jenkins, Jayashree Iyer, James Cousens, Rebecca Engelke, Peter Hsi, Rebecca Christianson, Elizabeth Wiellette

**Affiliations:** Draper, Cambridge, MA, United States

**Keywords:** 3D cell culture, dendritic cell, electrospinning, *in vitro* immune model, intestinal cell culture

## Abstract

**Introduction:**

Detailed research into intestinal immune exposure to luminal contents, including nutrients and microbial metabolites, could be well supported by accurate and reproducible models of the human intestinal milieu. Animal models provide insights but are not fully predictive of human biology, while human correlative studies are hampered by the complexity of feedback signals from established immune reactions. Therefore, characterization of human nutrient uptake and immediate effects on intestinal health will benefit from *ex vivo* models that provide controlled experimental conditions and include relevant human cells.

**Methods:**

This work introduces a novel platform that integrates key human immune cell types with intestinal epithelial cells (IECs), which provide both the barrier and the permissive interface between intestinal contents and immune cells. Specifically, cells from the small intestinal ileum are incorporated because this is a crucial site of immune system exposure, where Peyer’s patches of the epithelial lining provide a permeable interface for luminal content sampling by sentinel dendritic cells (DCs) and macrophages. To support relevant cell–cell contact and co-culture, we have built and evaluated a custom three-dimensional matrix similar to the lamina propria and designed to support DCs and T cells in direct contact with the IECs. The material is produced by electrospinning polycaprolactone into a bilayer material with two pore types: small pores to support epithelial organization into a tightly organized monolayer that provides appropriate barrier function and large pores that provide access and movement for the large, migratory DCs.

**Results:**

As a result of cell co-culture on the bilayer material, the two cell types interact directly with each other in a manner more similar to *in vivo* biology. This structure enables an *in vitro* model where nutrients, commensal microbes, pathogens and other antigens can be introduced to the epithelium, and the innate and adaptive immune response can be evaluated.

**Discussion:**

This platform has the potential to provide a consistent and reproducible context for studies of nutrient effects on human intestinal health and inflammation.

## Introduction

**Figure 1:**
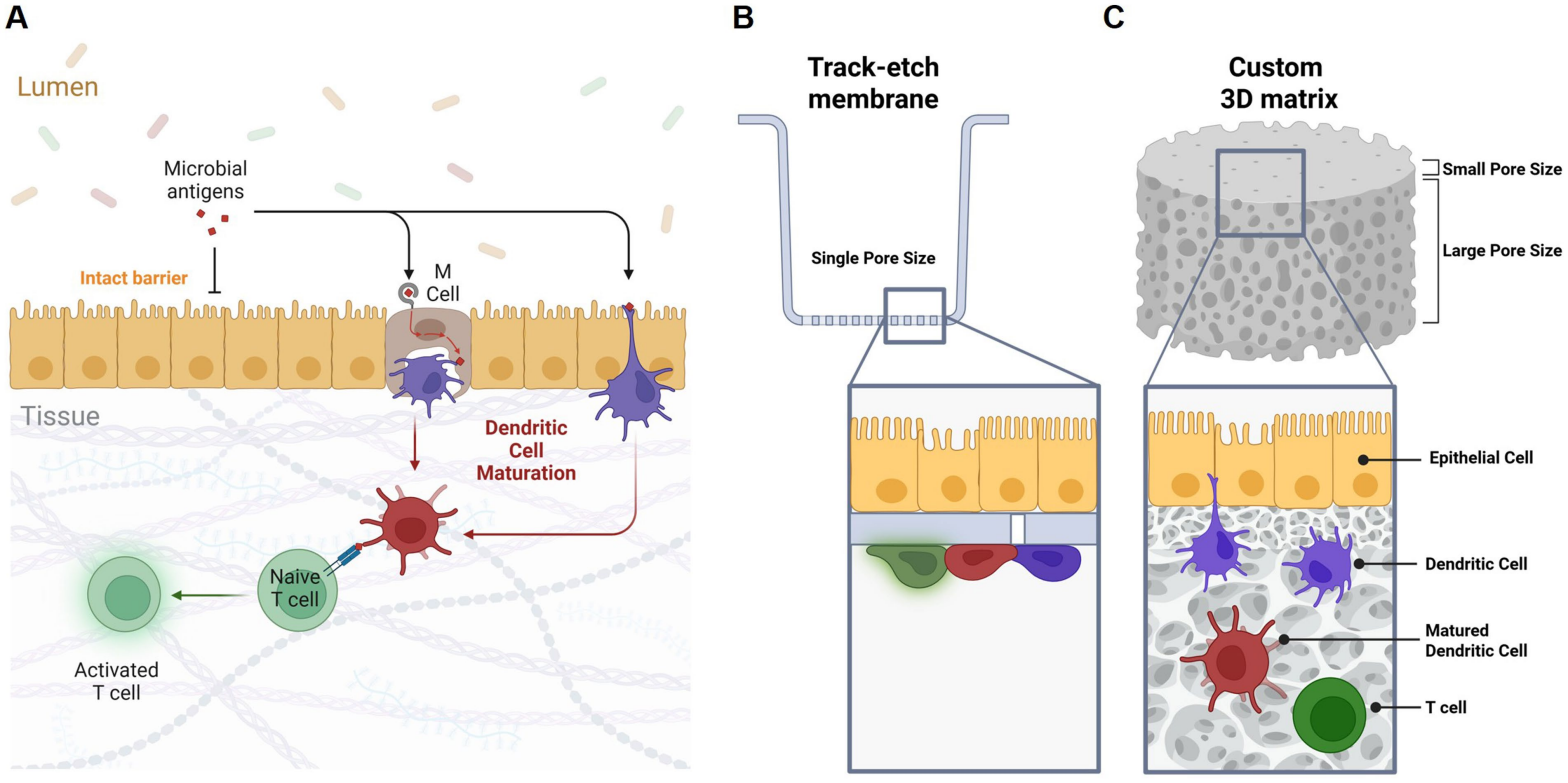
**(A)** Overview of cell–cell interaction between IECs and immune cells. **(B)** Two-dimensional *in vitro* model compared to **(C)** hypothesized electrospun three-dimensional matrix. Created in BioRender. Walker, J. (2025) https://BioRender.com/3n3j10n.

The human intestine supports a diverse range of functions, from uptake of nutrients and water to a range of neurological, muscular and immune activities. In fact, the intestine is the largest immune organ in the body and the site of extensive immune training. Controlled exposure of the immune system to antigens results in tolerance to food and microbiome as well as appropriate activation in response to pathogens (1–4). Regulated exposure is driven by the epithelium, which lines the lumen and creates a barrier between the external world (the lumen) and internal tissues (5, 6). Regulated breaks in this barrier occur at lymphoid tissues called Peyer’s patches in the ileum of the small intestine, where dendritic cells (DCs) and macrophages concentrate at the Peyer’s patches tightly associated with specialized epithelial microfold cells (M cells), which can actively take up antigens from the lumen (7, 8) (Figure 1A). Immature DCs and macrophages residing in the M cell “pocket” receive these antigens and present them to naïve T cells in nearby gut associated lymphoid tissues to initiate the adaptive immune response and inflammation (9). Immune tolerance versus inflammatory response are directed in part by these T cell responses (10–12).

To better understand and treat inflammatory responses to luminal contents, systems that accurately represent this biology are needed. Animal models are often insufficient to model immunologic response due to key differences with human biology (13). As an example, allergy modeling typically requires genetic modifications to Toll-like receptor 4, or the IL-4 receptor (14) to create sufficient sensitivity to support human-relevant experiments (15, 16). As an alternative, researchers have used human immortalized cell lines and primary cells for *in vitro* intestinal models, predominantly in the Transwell® platform (17–24). The Transwell® system consists of a hanging basket supporting a porous membrane that separates two chambers. Immune-epithelial co-cultures are typically constructed with epithelial barrier generated on the membrane and immune cells added to the other surface or bottom of the well; cell–cell communication is therefore limited to secreted factors or occasional trans-pore contact (25–30) (Figure 1B). These culture types are useful but limited in that they restrict the tissue architecture critical for the intestinal immune model by preventing immune cell trafficking into the intestinal layer.

We propose that a relevant *in vitro* model of human intestinal mucosal immunity should integrate the epithelial barrier with innate and adaptive immune cells, and that these cell types need to be co-cultured in a way that allows close contact between the cell types. We hypothesized that such a model would benefit from culture on a 3D matrix that is designed to support the specific culture needs of the intestinal epithelial cells (IECs) and the integrated immune cells while facilitating their physical interaction (Figure 1C). The primary challenge of this concept is balancing the different material requirements of the two different cell types. *In vivo*, the immune cells reside in the lamina propria that allows migration through three dimensions (31), while the epithelial cells require a supportive basement membrane to organize and orient the barrier monolayer (32).

Recently published models for epithelial-immune intestinal culture provide solutions to this challenge (33). A molded silk scaffold provides a sufficiently non-porous surface to grow primary epithelial monolayers as well as an underlying higher porosity scaffold that allows addition of support cells (34, 35). Another system uses a well insert with an open strut framework that holds a collagen hydrogel in place; the collagen filling the insert supports development of an epithelial monolayer with barrier function and still allows migration of immune cells through the collagen (36). At the same time these models were being developed, we started work on our system, with a focus on development of a material that would provide a low-porosity surface for epithelial growth and a highly porous matrix for migratory and 3D cell integration.

Herein we describe a platform for co-culture of intestinal epithelial monolayers and immune DCs comprising biocompatible polycaprolactone (PCL) electrospun to create a dual porosity, three-dimensional (3D) matrix with a dense layer to support epithelial culture and a more porous layer to support DC migration. Importantly, we correlated material properties across a range of material variants with key aspects of cell function, specifically epithelial monolayer barrier function and DC integration. We determined that low porosity and pore size are key determinants of epithelial barrier formation, while high open area supports DC movement through the material. This electrospun material provides a more physiological interaction between the two cell types, resulting in a relevant antigen response by the immune cells in the context of the epithelial barrier. Our concept for this system was founded in a desire to address food allergies systematically with human cells, however the constraints of working with immune cells of unknown exposure history during this proof of concept led us to rely on the endotoxin lipopolysaccharide during these proof-of-concept studies. The protocols presented here rely on primary human cells derived from ileal epithelium and immune cells derived from leukopaks; we have shown that a common donor is not required to support tolerant co-culture of the cell types. The use of primary cells also highlights the opportunity to integrate cells from specific donors, including those with established allergic sensitivities. Future uses of the platform may include studying enteric pathogen engagement with the host immune system and aspects of food allergy initiation.

## Methods

### Electrospun PCL mat fabrication

*Preparation of solutions for electrospinning*: All materials were purchased from Sigma-Aldrich unless otherwise stated. Solutions were prepared using 3:1 dichloromethane (DCM)/*N*, N-dimethylformamide (DMF) as the solvent phase. The 15% w/v solution was prepared by adding 1.5 grams of 80 kDa polycaprolactone (PCL) into a glass container, followed by the DCM/DMF mixture (10 mL). The 20% w/v solution was prepared by adding 2.0 grams of PCL into a container, followed by the DCM/DMF mixture (10 mL). For both solutions, the containers were capped and stirred overnight.

*Electrospinning*: Electrospun materials were prepared via electrospinning techniques using a climate-controlled apparatus (EC-CLI IME, The Netherlands) with a rotating target collector (EM-RTC, IME, The Netherlands). The temperature was maintained at 25 °C and the relative humidity was maintained at 60% for 30 min prior to electrospinning. Solutions were introduced using 30-mL syringes (BD) via syringe pump (IME), and electrospun fibers were collected onto a rotating drum collector that was wrapped in aluminum foil. The solution dispensing rate for the solution was 2 mL/h, and the collector spin rate was set to 15 rpm with a 75 mm distance to collector. Electrospinning needles were 1 mm in diameter with 0.33 mm inner wall thickness. The voltage for the collecting drum was set to −4 kV. The dispensing voltage was typically 14–18 kV, where the specific voltage for each run was selected during the run based on Taylor cone consistency. In cases of spinning multilayer mats, a syringe was loaded with a 20% w/v PCL solution. Once electrospinning of the first solution was complete (variable dispense volumes were used at a consistent dispense rate of 2 mL/h, Table 1), a separate syringe was loaded with a 15% w/v PCL solution, and the electrospun fibers were spun on top of those deposited using the 20% w/v solution. (Note: air bubbles were removed from solutions prior to dispense).

**Table 1:** Characterization of electrospun PCL material across 8 iterations and comparison to commercially available track-etched membranes.

Material	Fraction 20% PCL (Volume solution, mL)	Fraction 15% PCL (Volume solution, mL)	Thickness (μm)^α^	Bulk Tensile Modulus (MPa)^β^	Mean Pore Area (μm^2^)^#^	Open area (%)^#^	Bulk Porosity ^θ^	Fiber diameter (15% side, μm)	Fractal dimension (2-Δdim)	Epithelial monolayer	Immune integration
COTS 0.4 μm	N. A.	N. A.	20	2151.58 ± 1434.22	0.1 ± 0.0	6.0 ± 0.3	11.61 ± 0.03	N.A.	N.D.	**Yes**	No
COTS 3 μm	N. A.	N. A.	20	1411.67 ± 10.07	4.4 ± 1.1	7.0 ± 0.9	29.01 ± 0.10	N.A.	N.D.	No	**Yes**
DL01	0.54 (7)	0.46 (6)	186 ± 35	44.95 ± 5.19	5.7 ± 2.6	14.3 ± 5.7	53.80 ± 0.10	1.14 ± 0.80^Σ^	0.138	**Yes**	No
DL02	0.5 (7)	0.5 (7)	200 ± 27	81.63 ± 7.49	4.1 ± 0.8	4.8 ± 1.2	51.88 ± 0.87	1.29 ± 0.00^Σ^	0.113	**Yes**	**Yes**
DL03	1.0 (7)	0.0 (0)	68 ± 4	15.41 ± 6.79	3.5 ± 0.7	14.5 ± 0.6	58.97 ± 0.37	1.10 ± 0.46^Σ^	0.103	No	**Yes**
DL04	0.7 (7)	0.3 (3)	153 ± 17	35.13 ± 21.74	3.2 ± 1.0	10.3 ± 2.3	54.61 ± 0.54	1.16 ± 0.48^Σ^	0.113	No	**Yes**
DL05	0.125 (2)	0.875 (14)	297 ± 34	27.68 ± 15.77	5.2 ± 1.7	13.0 ± 2.4	65.65 ± 1.61	1.91 ± 0.59^•^ 0.36 ± 0.10^•^	0.188	No	No
DL06	0.5 (7)	0.5 (7)	212 ± 16	11.01 ± 3.24	2.4	4.8	58.84 ± 0.15	1.41 ± 0.00^Σ^	0.200	**Yes**	No
DL07	0.5 (7)	0.5 (7)	244 ± 26	6.53 ± 7.07	1.8	7.4	56.11 ± 0.44	1.61 ± 0.59^•^ 0.30 ± 0.04^•^	0.159	**Yes**	No
DL08	0.5 (7)	0.5 (7)	257 ± 19	23.41 ± 11.29	1.9	5.8	59.74 ± 0.35	1.44 ± 0.66^•^ 0.50 ± 0.16^•^	0.141	No	**Yes**

^α^Thickness data reported as average and standard deviation from nine measurements (wherein 3 independent measurements were taken from 3 different mat segments).

^β^Average and standard deviation of 3 measurements.

^#^Analysis on 20% PCL side of custom material and superior side of COTS.

^θ^Gravimetric Measure, 1 – (density_sample_ / density_material_), average of 3 measurements.

^Σ^Data collected using GIFT, an ImageJ macro optimized to analyze fiber diameter of electrospun mats from SEM images (15% PCL side).

^•^Images collected via SEM were not appropriate for use with the GIFT macro due to a bimodal distribution of fiber diameters within the mat. Ten large fibers and 10 small fibers were measured manually using ImageJ and averaged to report a “large” fiber and “small” fiber average for these mats. Examples indicating which images are and are not appropriate for GIFT macro use are shown in the SI.

N.A., not applicable.

N.D., not determined.

Bold values are to emphasize success with material to enable epithelial monolayer formation or immune integration.

### Characterization of electrospun PCL Mats

*Scanning electron microscopy (SEM)*: SEM imaging was performed using a Zeiss Supra 35VP model with S/N 2422 and Zeiss software Smart SEM version 6.00. Samples were cut from bulk electrospun mats at 0.75 cm × 1.5 cm in size, and mounted onto samples holders using carbon tape. Once mounted, samples were gold sputtered (Au,99.99%) in a Cressington sputter coater for 45 s. Images were taken using a Zeiss SEM at 10 kV for magnifications of 50x, 250x, 500x, 1,000x, and 2,500x. To obtain 90 °cross sections, samples (typically 2.5 cm × 2.5 cm) were submerged in liquid nitrogen and cut with metal scissors. Note that the metal blades were also submerged in liquid N2 prior to making the cut. A 4.5 cm × 5.5 cm flat fixture with two spring loaded block edges was used to hold cross section samples for imaging.

*Dynamic mechanical analysis (DMA)*: Samples were run on a TA Q800 DMA, using the thin film fiber clamp. Samples sizes were ca. 20 mm × 6 mm × 0.21 mm, with exact sizes (calipers/ruler) entered as a parameter prior to each measurement. Samples were attached and set up to 0 N of preload force. The samples were equilibrated at 37 °C and held under isothermal conditions for 5 min. The length of the sample was measured before ramping the force up to 0.500 N at a rate of 0.05 N/min.

*Gravimetric analysis*: Gravimetric analysis data for each mat was collected on square coupons of approximately 20 × 20 mm in size. Each individual coupon was measured for mass on an analytical balance, length and width across using a standard ruler, and height using a set of Mitutoyo Digimatic calipers. Each measurement was performed in triplicate at different points upon the same coupon to provide an average value with uncertainty.

Porosity was further derived from this data by dividing the mass of each coupon by its volume (calculated as length × width × height) to yield its density. The ratio of coupon density to reported (manufacturer, Sigma Aldrich) PCL density (1.145 g/cm^3^) was subtracted from 1 to yield the amount of empty space (i.e., porosity) within the sample.

This method was validated by performing analogous measurements on commercially available track etched membranes with manufacturer reported porosity data. Polycarbonate (it4ip, 3 μm pores, 22 μm thick, 1,000M25/610M303/293) had a manufacturer reported porosity of 21.2% porosity and was measured at 29% porosity using the gravimetric analysis method. PET (it4ip, 0.4 μm, 12 μm thick, 2000M12/710 N403/293) had a manufacturer reported porosity of 12.6% porosity and was measured at 11.6% using the gravimetric analysis method.

*Determination of fractal dimension*: SEM images taken at magnification of 1,000X were first processed using *imbinarize* (Matlab, Image Processing Toolbox) and then analyzed for fractal dimension using the previously reported box-count method (37, 38).

*Fiber diameter analysis*: Average fiber diameters were collected using GIFT, an ImageJ macro optimized to analyze fiber diameter of electrospun mats from SEM images (15% side). For some mats, images collected via SEM were not appropriate for use with the GIFT macro due to a bimodal distribution of fiber diameters within the mat (Supplementary Figure 1). In such cases, 10 large fibers and 10 small fibers were measured manually using ImageJ and averaged to report a “large” fiber and “small” fiber average for these mats. This is representative of a previously reported issue where it was found that woven mats of varying fiber thicknesses tend to result in inaccurate diameter readings (39). The inability of the macro to map such a bi- or multi-modal distribution causes the Gaussian to average multiple peaks or identify only one of many. We posit that mats DL05, DL07, and DL08 had a more significant quantity of both thick and thin fibers compared to the other successful mats, leading to an error in the final average fiber diameter presented by the macro (40).

### Device assembly

*3D-Printed housing*: The device housing prototype was designed in SolidWorks and 3D printed (Form 2, Formlabs) using either Dental SG or ULTEM. The housing design features three gaps between evenly spaced prongs which allow for both STX2 and STX4 trans-epithelial electrical resistance probes to fit comfortably to assess the epithelial monolayer health (World Precision Instruments Inc., Sarasota, Florida). The device housing presented here is compatible with a 24-well cell culture plate and features a growth surface area of 0.33 cm^2^, however the 3D printed design can easily be adjusted to accommodate other plate formats. The circular bottom rim of the 3D printed housing structures was scored with 240 grain sanding paper. The devices were rinsed using DI water, sonicated for 30 s in cold 70% ethanol, and air-dried. Design files are available from the authors upon request.

*Adhering electrospun matrix to housing*: The housing structures and electrospun mats were joined with medical device adhesive UV curable glue (1180-M-Gel, Dymax Corporation, Torrington, CT). A thin layer of glue was applied to the circular bottom rim of the housing, and the housing was pressed onto a 10 mm diameter cutout of the electrospun material using a biopsy punch (Medex Supply, Passaic, New Jersey). Pressure was applied for 60 s while a LED-200 UV gun was used to cure the glue (Thorlabs, Newton, New Jersey). The device was then placed under sterilizing UV light for 15 min for final cure.

### Cell culture

*Primary intestinal epithelial spheroid culture*: Isolated primary ileum IECs are maintained as previously described (41) and passaged weekly. Spheroids were cultured in 3D Matrigel (Corning, REF356255) domes and supplemented with proliferation media (PM; formulated as previously described (42)) and exchanged every other day. Upon passaging, spheroids were broken down using 0.25% Trypsin followed by mechanical agitation and centrifugation (400 g for 4 min). The cell pellet was resuspended on ice in undiluted Matrigel and replated in 3D domes, which were then polymerized at 37 °C, before submerging in PM and culturing at 37 °C with 5% CO_2_.

*Immune cells isolation*: Primary human monocytes and primary human T cells were magnetically isolated from human leukopaks (Charles River) using the CD14 microbead and Pan T kits (Miltenyi, 130-050-201 and 130-096-535, respectively) according to manufacturer’s protocols. Both collections were frozen separately at 20e6 cells/mL in Recovery™ Cell Culture Freezing Medium (Thermo Fisher) and stored in liquid nitrogen.

*Dendritic cell differentiation, induced maturation, and migration*: Monocytes were thawed and cultured in base media (RPMI 1640, Gibco, 61870-036) with 10% HI-FBS and supplemented with GM-CSF (50 ng/mL, Pepro Tech Inc., 200-04) and IL-4 (25 ng/mL, Pepro Tech Inc., 200-04). Differentiation of monocytes into immature DCs was achieved after 5 days, with fresh media supplemented every 2 days. Maturation of DCs was induced through LPS exposure (100 ng/mL, Sigma, L8274-25MG) for 24-h. Differentiation and subsequent maturation were characterized by flow cytometry. Chemoattraction of matured DCs (mDCs) was induced with CCL21 (200 ng/mL, PeproTech, 300-35A), a chemokine with established capability driving DC migration *in vitro* (43). In the case of migration assays, mDCs were seeded into the top chamber of the Transwell® baskets with 0.4, 3, or 8 μm pore diameters. Bottom chamber media was supplemented with CCL21 or not, and after 4 h the contents of the bottom chamber were collected and counted.

*T cell culture and induced activation*: T cells were thawed and cultured in base media (RPMI 1640, Gibco, 61870–036) with 10% HI-FBS and supplemented with IL-2 (100 ng/mL, Milteni Biotec, 130-097-743). Media was refreshed every 2 days. T cell TransAct, human (Miltenyi, 130-111-160) was used to induce activation and activation status was characterized by flow cytometry.

### Epithelial-immune tissue model in electrospun matrix devices

*Device treatment prior to seeding*: Devices were treated with oxygen plasma (March Asher) for 30 s and 100 W to increase the hydrophilicity of the electrospun material, then soaked in 70% ethanol for 5 min. Devices were then washed 3 times with PBS and incubated in media overnight at 37 °C to remove any residual ethanol.

*Primary intestinal epithelial cell seeding and culture*: Media was aspirated from devices and are then placed in a bath of Matrigel (diluted 1:40 with ice cold PBS) that submerges the electrospun material to coat both the top and bottom surfaces. Devices were incubated at 37 °C for 60 min and the ECM aspirated. Attachment factor (Cell Systems, 4Z0-201) was warmed to 37 °C and added to each device and immediately aspirated. IECs were seeded in PM at 0.8 × 10^6^ cells/mL into the inner cup of each device, onto the top, 20% w/v PCL side of the electrospun material. The devices were transferred to a 24-well plate and PM was added to the well for each device. The following day, fresh PM was added to the cup and the well of the devices and completely refreshed 48-h after seeding.

*Primary intestinal epithelial cell differentiation*: Four days after seeding, PM was exchanged for differentiation medium (DM; formulated as previously described (42)). DM was supplemented with RANK-L (100 ng/mL, Peprotech, 310-10) for the first 3 days of differentiated culture (days 4–7). On day 7, devices were gently rinsed with PBS to remove residual RANK-L and cultured in DM media without any supplementation. Media was refreshed every other day.

*Immature dendritic cell seeding and culture*: Monocyte derived immature DCs (iDCs) were resuspended at 14 × 10^6^ cells/mL in IEC Differentiation Media (DM). Media was gently aspirated from the devices and devices were transferred to a 6-well plate and gently rested upside-down. The iDC cell solution (50 μL) was pipetted onto the exposed bottom side of the device and cultured inverted at 37 °C with 5% CO_2_. After 2 h, devices were reverted and transferred to a 24-well plate. DM was added to both the cups of the devices and the well and returned to incubation at 37 °C with 5% CO_2_ for at least 4 h without disturbance.

### Characterization of tissue model

*Trans-epithelial electrical resistance (TEER)*: Trans-epithelial electrical resistance (TEER) measurements were taken to quantify barrier function of the IECs before and after co-culture with immune cells. An Electronic Volt/Ohm meter and electrodes were utilized to measure TEER (EVOM2, World Precision Instruments Inc., Sarasota, Florida). Each experiment included a blank Transwell® (Corning) or custom device. These contained culture media with no cells and were utilized to record a baseline, blank TEER value alongside experimental conditions. Prior to reading TEER, the cell culture plate was placed in a biosafety cabinet for 15 min to allow the temperature to equilibrate to room temperature. Tissue TEER (*Ω* cm^2^) for each device was calculated by subtracting the blank resistance of the membrane or scaffold from the resistance measured each day. This resistance was then multiplied by the surface area (~0.33cm^2^).

*Permeability*: Fluorescently conjugated 10,000 MW Dextran (0.5 μg/mL, Cascade Blue™, Invitrogen, D1976) was dosed to the apical cup of devices. After 24 h of incubation, media was collected from the apical and basolateral sides of the devices. Fluorescent intensity of the media was measured using the Synergy H1 (Bio Tek) with excitation at 400 nm and emission collected at 420 nm. Dextran-Cascade Blue™ concentration was determined against a standard. Permeability across the endothelial monolayer was determined by dividing the concentration of dextran in the basolateral side of the device and normalizing it to the concentration across the same acellular membrane, shown as Percent Transfer Relative to Blank. Statistical significance was determined by *t*-test.

*Live cell staining*: Live cells were stained using CellTracker Fluorescent Probes (ThermoFisher, C34565) according to vendor protocol. DCs were stained with Cell Tracker Green and IECs were stained with Cell Tracker Red. Each cell type was washed in PBS before introducing to co-culture. T cells stained with CellTrace CFSE Cell Proliferation Kit (ThermoFisher, C34554) according to vendor protocol.

*Immunostaining*: Electrospun scaffolds were briefly rinsed in cold PBS before fixing with fresh cold 4% PFA at RT for 10 min. Fixation was followed by 3 PBS washes at 5 min each before material was cut out with a scalpel. Extracted mats were placed in a 24 well plate so that each staining solution completely immersed the material. Monolayers were permeabilized with 0.3% Triton X-100 (Sigma, T8787) for 10 min, followed by 3 PBS washes at 5 min. Blocking occured subsequently with 3% normal goat serum (NGS), incubated overnight at 4 °C. Primary antibodies: ZO-1 (ThermoFisher, 33-9100), GP2 (MBL, D27-3), SpiB (Cell Signaling, 14323) were diluted 1:200 in blocking buffer and were also incubated overnight at 4 °C. The next day, 3 PBS washes were performed at 5 min each prior to secondary incubation. Pre-conjugated antibodies and stains CD86-AF647 (BioLegend, 305416), CD11c-FITC (BioLegend, 337214), Hoescht (ThermoFisher, H3570), and Phalloidin (Abcam, ab176759) as well as secondary antibodies were diluted in 3% NGS at 1:100, 1:100, 1:500, 1:1,000, and 1:300 respectively, and incubated overnight at 4 °C. Scaffolds were washed in PBS and mounted with Vectashield (Vectorlabs) on a microscope slide, and sealed with a coverslip for confocal laser scanning microscopy.

*Microscopy*: Live and fixed samples were imaged on a confocal microscope (Carl Zeiss, LSM-700). Images were processed and analyzed using ImageJ (44).

*Flow cytometry*: Live cells were resuspended and washed in MACS Buffer + 0.1% BSA three times then incubated in FC Block (1:50, Biolegend, 422302) for 15 min at room temperature. Cells were labeled with antibodies all at 1:200 dilution: CD14-FITC (Biolegend, 325604), CD11c-PerCP eFluoro-710 (Invitrogen, 46-0116-42), CD83-APC (Biolegend, 305312), and CD86-SB-600 (Invitrogen, 63-0869-42), and incubated for 30 min at 4 °C. Samples were incubated in Sytox Blue or Sytox Green (1:1,000, ThermoFisher, S34857 and S7020, respectively) for 5 min at room temperature. Cells were washed and analyzed or fixed in 4% PFA for 10 min, washed, and stored at 4 °C. When cells were to be fixed, they were first co-incubated with Zombie-NIR (1:1,000, Biolegend, 423,105) alongside FC block. Cells were analyzed using an AttuneNxt (Thermo Fisher) flow cytometer and data was processed using FlowJo (BD Biosciences, version 10.7.2 – version 10.10). Gating was set using Fluorescence-minus-one. After removing doublets, live cells were first gated either Sytox^−^ or Zombie^−^. DCs were identified by CD11c^+^ and mDCs further gated by CD86^+^. T cells were identified by CD3^+^ and further gated into activated T cells by CD69^+^, CD25^+^. For T cells stained with CFSE, daughter cell generations were determined by peaks descending in fluorescent intensity.

*Statistics*: Data was analyzed using Prism (GraphPad). Data are shown as mean ± s.d. for a given number of biological replicates. Significant differences were defined by *p* < 0.05 for all statistical methods. Differences between the experimental groups were analyzed by unpaired, two-tailed Student’s *t*-test, or one-way or multi-way analysis of variance (ANOVA). *Post hoc* pairwise analysis was done using Tukey’s HSD test.

## Results

### Co-culture of primary epithelial and immune cells

**Figure 2:**
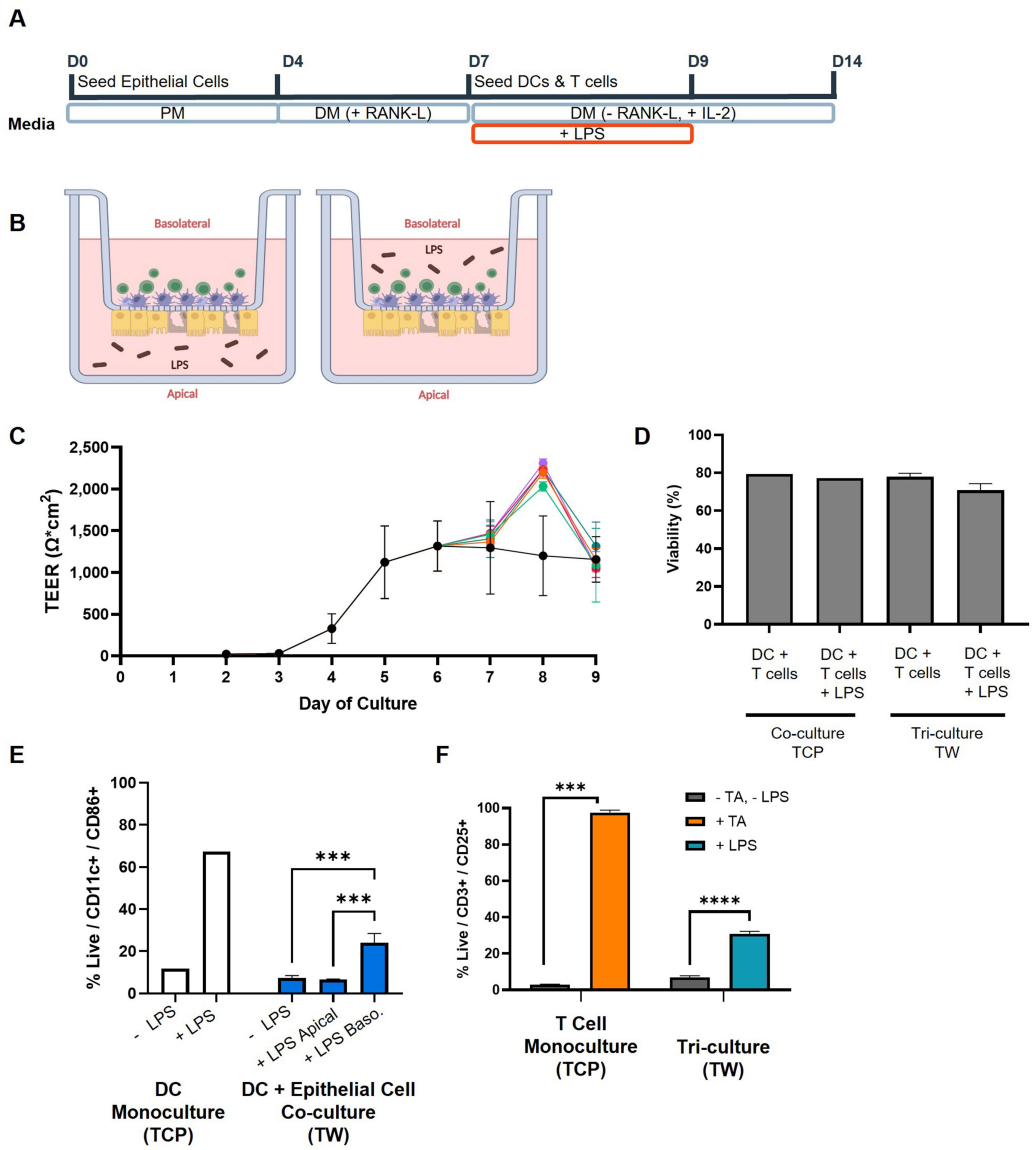
Tri-culture on commercial Transwell® inserts requires direct LPS exposure on basolateral side of model to direct DC maturation and T cell activation. **(A)** Experimental timeline and **(B)** schematic of epithelial-immune co-culture utilizing commercial TW inserts. **(C)** TEER measurements of epithelial tissue under different co-culture conditions (black – IEC alone, *n* = 4; red – IEC + unstimulated DCs *n* = 3; green – IEC + DC + T cells *n* = 5; blue – IEC + DC + apical LPS *n* = 5; purple – IEC + DC + basolateral LPS *n* = 3; orange IEC + DC + T cell + basolateral LPS *n* = 5) **(D)** Flow cytometry-derived immune cell viability at day 14 of culture with immature DCs (iDC), T cells, and LPS cultured together in tissue culture plastic (coculture TCP, *n* = 1) or with IEC monolayers in TW inserts (Tri-culture TW, *n* = 5). **(E)** Flow cytometry-derived measurement of DC maturation measured as the CD86^+^ subset of live CD11c^+^ cells. Treatment of DCs grown in monoculture in tissue culture plastic (TCP) *n* = 1 or in culture with IECs in TW inserts *n* = 4 with or without LPS treatment apically (IEC side of insert) or basolaterally (DC side of insert). Statistical significance by One-Way ANOVA, post-hoc Dunette’s, ** = *p* < 0.01. **(F)** Flow cytometry-derived measurement of T cell activation measured as the CD25^+^ subset of CD3^+^ cells after exposure either direct activation with Transact (TA) (Monoculture TCP) or to DCs grown with IECs and stimulated with LPS (Tri-culture TW), measured at day 14 of culture program. Statistical significance determined by unpaired *t*-test within mono- and tri-cell culture conditions (*n* = 3), *** = *p* < 0.001, **** = *p* < 0.0001. Created in part in BioRender. Walker, J. (2025) https://BioRender.com/3n3j10n.

We first developed protocols in the Transwell® platform to establish media and timeline conditions required for successful co-culture of human primary epithelial and immune cells. Primary human IECs were established on the bottom of the membrane in PM media and allowed to proliferate for 4 days, at which point the media was changed to differentiation media (DM) supplemented with RANK-L (Figure 2A). After an epithelial cell monolayer was established, as determined by TEER, we introduced DCs and T cells; these were isolated from the same donor, distinct from the epithelial cell donor (Figure 2B). Human monocyte-derived DCs were characterized as immature based on CD11c^+^; CD83^lo^, CD86^lo^, HLA-DR^lo^ (Supplementary Figure 2) and were seeded at day 7 with T cells (CD3^+^) onto the basal side of the epithelial monolayer in DM. DM alone did not induce substantial maturation of DCs (Supplementary Figure 3). In order to model luminal exposure to pathogenic bacteria, lipopolysaccharide (LPS) was added to the apical side of the epithelial cells. As a positive control, LPS was added directly to the immune cells located on the basal side of the epithelial monolayer. LPS was used as a surrogate for allergens at this point; immune cells isolated from deidentified donors had unknown antigen exposures and sensitivities and so we focused on a universal stimulant of immune reaction for this proof-of-concept stage.

TEER was used to monitor the intestinal epithelial barrier function throughout culture. High TEER (>1,000 Ω*cm^2^) was achieved following differentiation of the ileum monolayer and maintained through day 9 in unperturbed control monolayers (Figure 2C). Addition of immune cells on day 7 resulted in increased TEER, likely resulting from the additional cell density, however, the TEER consistently recovered back to the level of epithelium alone by day 9. This recovery was not the result of immune cell death because these cells were recovered and cultured for an additional 5 days with evidence of cell viability (Figure 2D). Immune cell activation was evaluated by flow cytometry 5 days after removal of cells from the tri-cell culture with 2 days of LPS exposure. LPS applied directly to DCs grown in well plates can mature the cells such that almost 70% of the DCs are CD86^+^ (Figure 2E). When LPS was applied directly to DCs grown in culture with IECs, about 15% of the DCs were CD86^+^ (Figure 2E). However, when LPS was applied on the apical side of the epithelial monolayer, no distinguishable DC maturation was identified (Figure 2E). We theorized that these data indicate that the immune cells were unable to sample LPS through the membrane and epithelial monolayer in this system. T cells were activated in response to LPS-induced maturation of DCs, where a significant increase in CD3^+^/CD25^+^ cells was identified in the presence of basolateral LPS addition compared to untreated cells (Figure 2F).

### Development of a custom electrospun material

**Figure 3:**
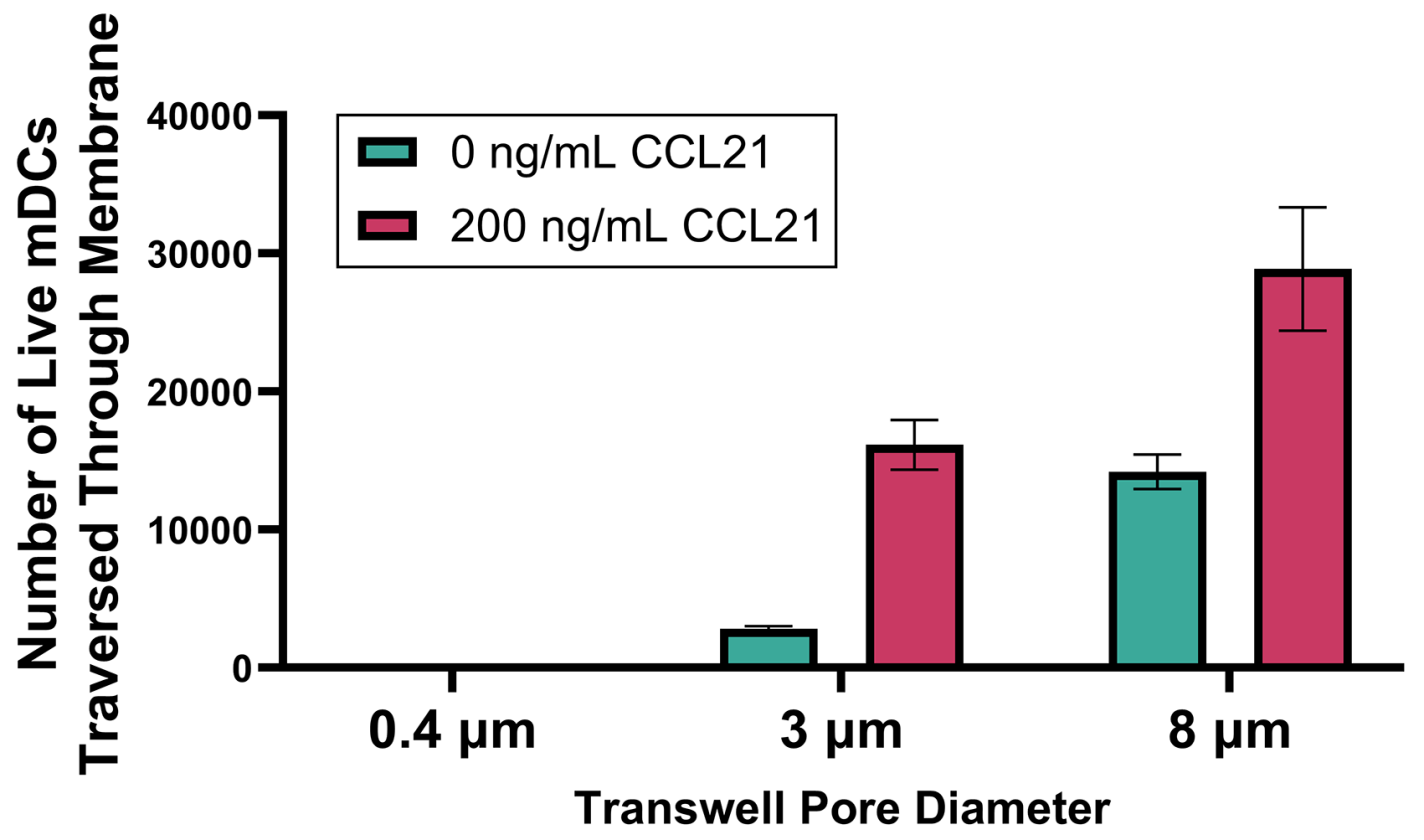
Transmigration of mature DCs (mDC) is restricted based on membrane pore size. The total number of mDCs that migrated through Transwell membranes of different pore sizes was counted. *n* = 3 independent Transwell® devices for each condition; error bars indicated standard deviation.

The experiments reported above were performed in TW devices that contained a 0.4 μm pore size, 1% porosity track-etched membrane, and we hypothesized that increasing DC access to the epithelial layer could support active immune sampling across this barrier *in vitro*. Supporting this idea, we found that DCs do not measurably migrate through 0.4 μm pores, even when CCL21 is present on the opposite side of the membrane to create a relevant chemokine gradient (Figure 3). Increasing pore size did allow transit of DCs, which was enhanced in the presence of CCL21 gradient (Figure 3). Therefore, we set out to develop a custom, dual porosity material with a semi-porous surface layer to support epithelial monolayers and a highly porous layer to allow immune cell movement. Electrospun polycaprolactone (PCL) was selected from among several methods and materials (45, 46), including colloidal templating (31) and cross-linked hydrogels (47). Electrospinning enabled tuning of the bulk material by modulating parameters during production, including voltage, distance to collector, solution flow rate, solution concentration, polymer molecular weight and environmental humidity (48). PCL is a common, biocompatible polymer, and has a degradation time on the order of years (49). Several studies detail relationships of electrospun PCL morphology and cell seeding behavior (50, 51). Electrospun PCL has been demonstrated as a successful material for the culture of cells (fibroblasts, keratinocytes) (45, 46, 51) as well as DCs (52, 53). We therefore settled on this material and method for this effort.

Electrospinning parameters for evaluated mats were chosen after empirical tests with varying solvents and PCL concentrations. Using larger percentages of a volatile solvent (methylene chloride) improved fiber formation, while decreased percentages of dichloromethane led to lower fiber formation and amorphous polymer deposition (data not shown). We determined that the 75:25 solution of methylene chloride/*N,N*-dimethylformamide (DMF) was optimal. We experimented with PCL concentrations from 10% w/v (weight/volume) through 20% w/v and performed studies to understand typical fiber morphologies across the range. Higher concentrations (>20%) proved too viscous for consistent dispensing. Low concentrations (10% w/v) resulted in beading (data not shown), presumably due to the lower viscosity leading to decreased stability in the electrospun fibers. It was found that with the temperature maintained at 25 °C and the relative humidity maintained at 60%, the 15–20% w/v range had the highest repeatable fiber production. After several iterations, we learned that the use of 20% w/v (weight volume) PCL resulted in fibrous mats with observably lower spacing between fibers whereas the 15% w/v PCL solutions resulted in stable fiber streams with observably more spacing between fibers. The observations were based on qualitative evaluation of SEM data.

**Figure 4:**
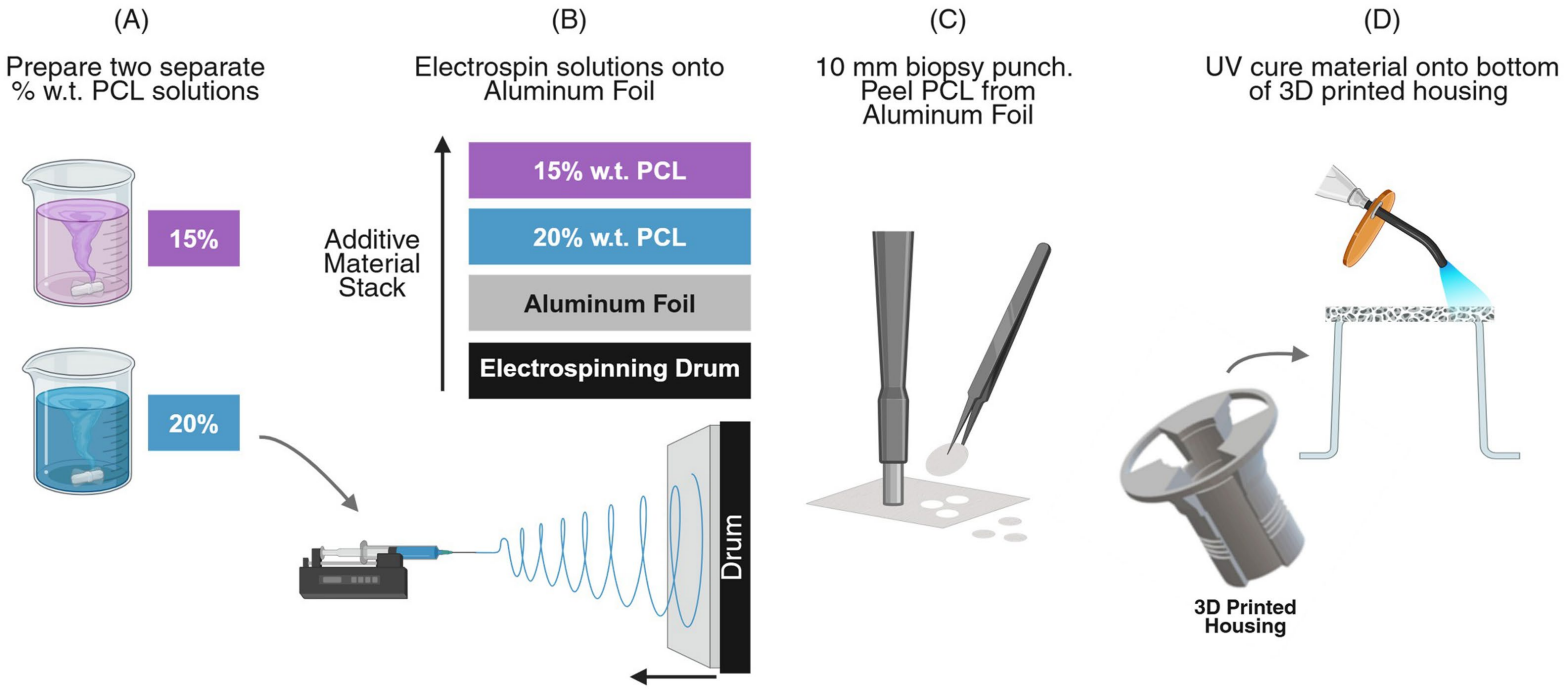
Process flow for electrospun 2D/3D devices. **(A)** Solutions of polycaprolactone (PCL) are prepared at appropriate concentrations (20% wt/vol and 15% w/v). **(B)** The PCL solutions are used to generate fibrous mats using electrospinning techniques. The electrospun fibers are spun onto a drum collector coated with aluminum foil. A higher viscosity solution (20% w/v PCL) is deposited first, followed by a lower viscosity solution (15% w/v PCL). **(C)** Biopsy punches are used to cut segments of the electrospun mats at appropriate sizes. The electrospun PCL mats are peeled from the supporting foil. **(D)** The electrospun mat segments are adhered to electrospun 3D-printed housing using a UV-curable glue. Created in BioRender. Walker, J. (2025) https://BioRender.com/3n3j10n.

The material process is shown in Figure 4, where the dual-phase material was achieved by spinning two layers of PCL at different concentrations, and therefore different viscosities, in a 75:25 solution of methylene chloride/*N, N*-dimethylformamide (DMF) at 20% w/v PCL followed by 15% w/v PCL. The higher viscosity 20% PCL solution was dispensed first and formed a flattened layer (Figures 4A, B) against the foil on the electrospinner collector; this first portion of the 20% PCL layer was distinct from the remainder of the spun mat, and it provided a flat surface to support epithelial cell culture. Analysis of pore size on the 20% surface (see below) characterizes this flat surface. After applying the 20% PCL solution, the lower viscosity 15% PCL solution was dispensed, forming a more porous layer (Figure 4A). We experimented with different ratios of the two solution volumes (Table 1) to generate electrospun mats with varying properties. Biopsy punches were used to cut circles of the electrospun material, and these cutouts were adhered to custom housings that provided a frame for suspending and manipulating the electrospun material (Figures 4C,D). The mats were adhered using a UV-curable, medical grade adhesive that is moisture resistant. The adhesive was stable to standard processing (70% ethanol for 5 min) and throughout all cell culture experiments.

Eight of the electrospun mats were evaluated for both physical properties and compatibility with DC integration and epithelial monolayer formation (Table 1). Typical methods were used to measure thickness, modulus and bulk porosity, noting that these methods cannot evaluate distinct contributions of the 15% vs. 20% w/v PCL layers (see Methods). Pore area, total open area, fiber thickness and fractal dimension were all derived by analyzing scanning electron micrograph (SEM) images collected from multiple areas across each electrospun material produced and therefore were unique to each face of the material (see Methods). It was challenging to measure the pore size or “openness” of the 15% w/v PCL material face since the three dimensionality of the material is flattened in two-dimensional image capture. Therefore, we developed an image analysis method that computed the fractal dimension via the box-counting method (38) (See Methods). The box-count dimensions can provide an approximate relative grouping of structures as more or less fractal-like. We define fractality as 2 minus the box-count dimension. In this analysis, a larger fractality indicates images with greater self-similarity of structure across multiple size scales. Therefore, a material with larger pores and higher porosity would have a lower fractality. The analyzed electrospun materials resulted in fractality ranging from 0.1 to 0.2 (Table 1).

**Figure 5:**
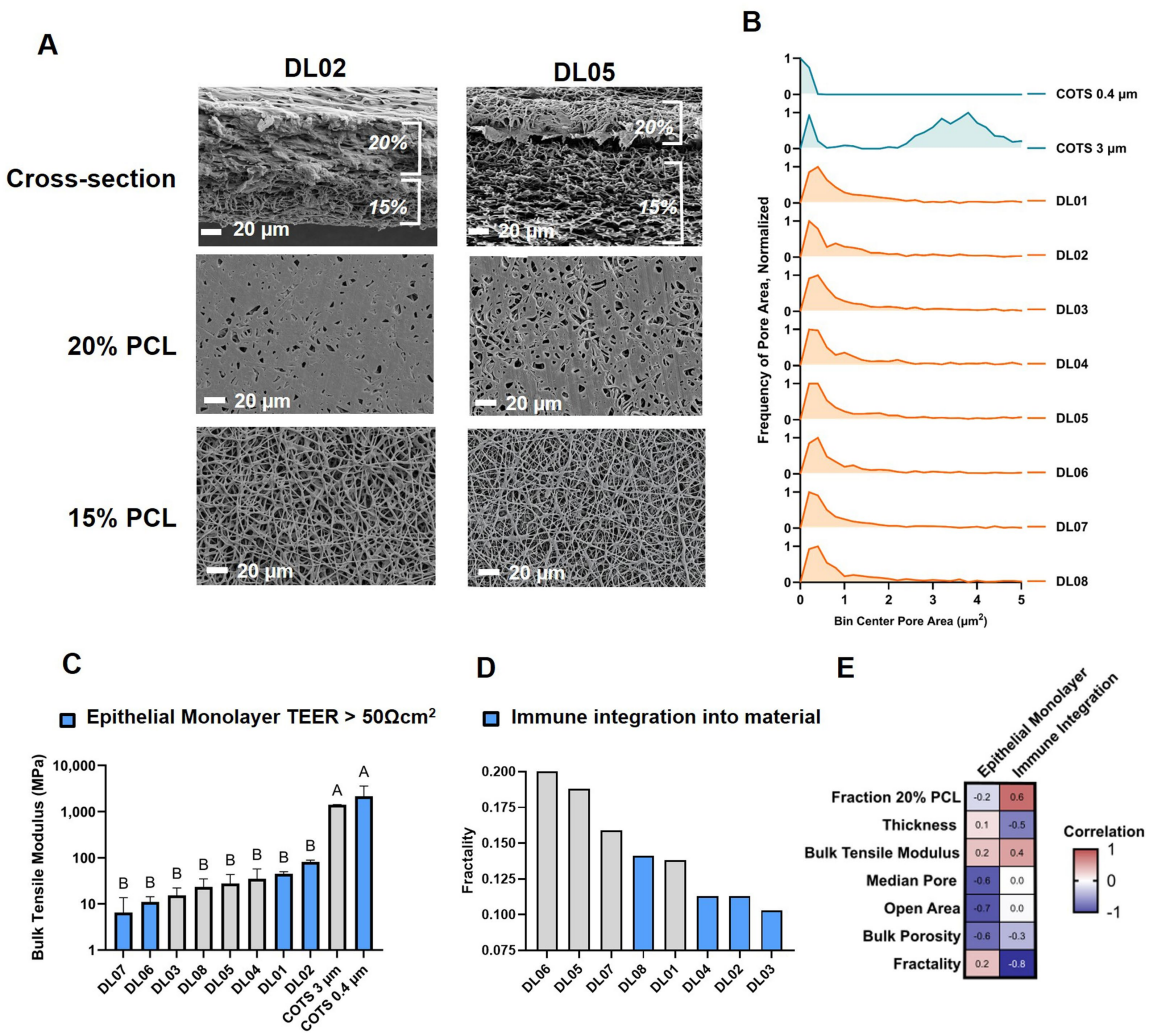
Characterization of electrospun mats. **(A)** SEM images of select electrospun materials. Scale bars represent 20 μm. The middle panel (20% PCL) represents the epithelial-facing side; the bottom panel (15% PCL) represents the immune-facing side. **(B)** Pore size area for 20% w/v PCL electrospun material (orange) and COTS 0.4 μm and 3 μm distribution (blue). **(C)** Bulk tensile modulus of electrospun materials. Material iterations with successful epithelial monolayer formation highlighted in blue, *n* = 3 separate devices/material. A and B labels indicate statistical distinction between modulus measurements. **(D)** Fractality of 15% w/v PCL side of electrospun materials. Fractality is defined as 2 minus the fractal dimension. Material iterations with successful immune integration are highlighted in blue. Image analysis was performed on one device/mat with *n* = 1–11 total images analyzed/mat. **(E)** Correlation matrix between material characterization and biological outcomes, either epithelial monolayer or immune integration.

SEM was used to visualize each product, and two example iterations, DL02 and DL05, demonstrated some of the variation observed (Figure 5A). Cross sections of the materials revealed the transition of fiber density from the electrospun 20 to 15% w/v PCL. The two examples also highlight visible differences in the total open area of both 20 and 15% PCL surfaces (Figure 5A). These observable differences were quantified for eight iterations of material production, DL01-DL08, by SEM imaging. The pore sizes on samples of each 20% w/v PCL face were analyzed for each material iteration and compared to two commercially available (commercial off the shelf, COTS) track-etched materials found in 0.4 and 3 μm Transwell® devices (Figure 5B). The majority of pores on the electrospun materials were within the size range of the 0.4 μm pore diameter COTS and below the 3 μm pore diameter COTS. Unlike the 0.4 μm COTS, the electrospun materials also had a right tailed distribution of larger pores. The increased open area on the epithelial facing side of the material may enable immature DC sampling of the apical side of the tissue model, which is physically blocked by the homogenous 0.4 μm pore COTS membrane.

To identify factors that influence the interaction of epithelial cells and DCs with the electrospun materials, we correlated physical features with successful epithelial monolayer formation, measured by TEER, and DC integration into the material matrix, determined by live microscopy imaging after 4-h of DC culture on the 15% w/v PCL electrospun material. We hypothesized that monolayer formation might depend on the modulus of the material. The modulus was very consistent across different material products, particularly when compared to the much stiffer membrane found in Transwells (Table 1; Figure 5C). Monolayer formation did not correlate with modulus, either within the range of moduli generated by custom materials or when compared to the COTS materials (Figure 5C). We also hypothesized that larger pores in the less dense material would better support introduction and penetration of DCs into the electrospun material. Successful DC culture correlates with pore size as measured by fractal dimension (Figure 5D). To identify the strongest correlates of preferred cell culture behavior, we associated overall monolayer formation (TEER) and DC integration with each of the physical properties and identified pore size as the key feature for each successful cell culture type (Figure 5E). Epithelial monolayer formation is most strongly counter-correlated with pore size, indicating that smaller pores are required to support monolayer formation. Successful incorporation of DCs is most strongly correlated with larger pore area and total open area, as well as counter-correlated with large fractal size; taken together these indicate a requirement for large, open pores to support DC movement into the material.

### Evaluation of cell culture on electrospun material

**Figure 6:**
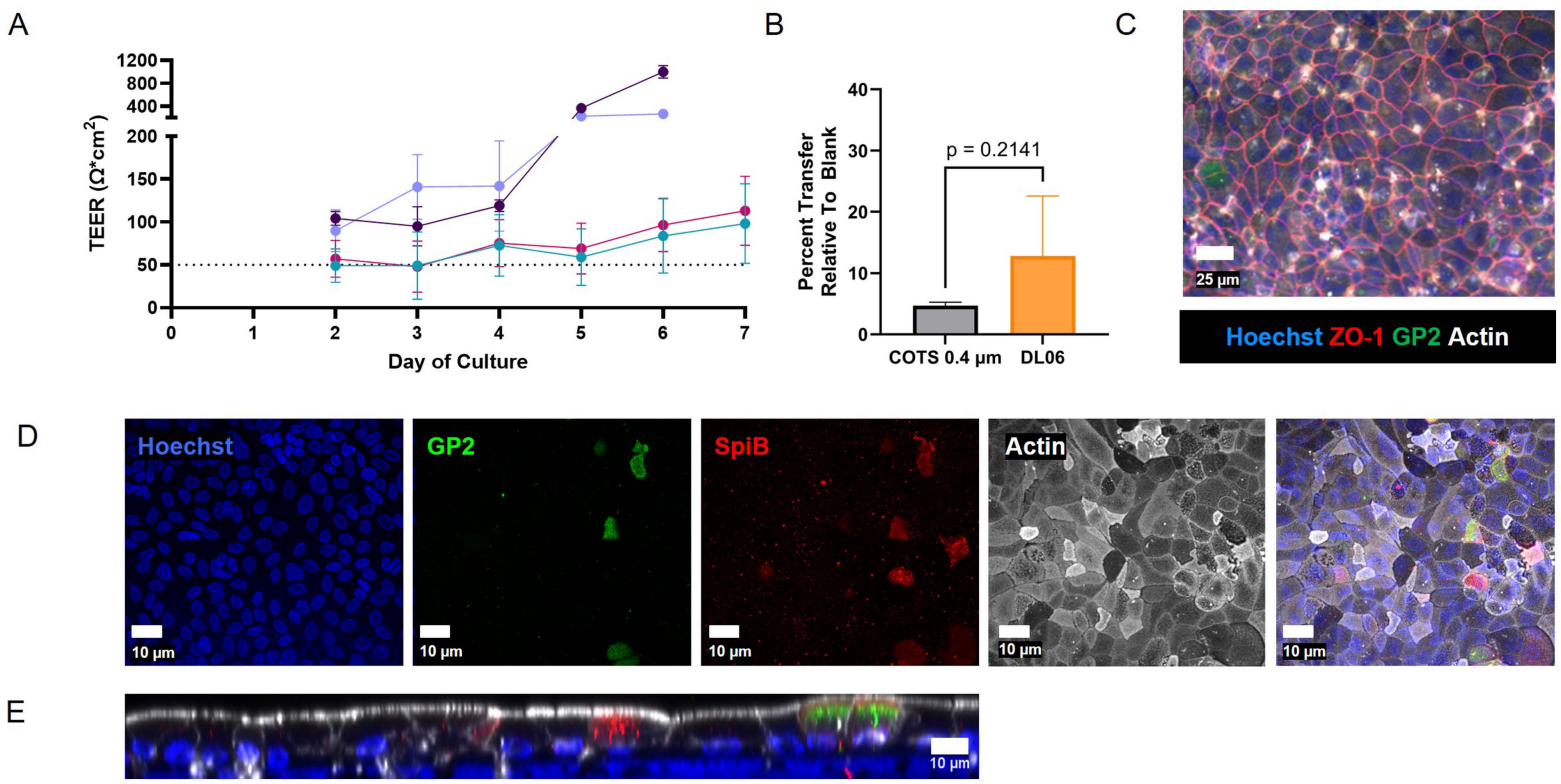
Epithelial cells form monolayers on electrospun PCL material with functional barriers and M cell differentiation. **(A)** TEER measured for IECs seeded on select electrospun materials that formed monolayers (dark purple – DL07, one experiment, *n* = 4; light purple – DL06, one experiment *n* = 4; pink – DL02, two experiments *n* = 4, *n* = 8; green – DL01, two experiments, *n* = 4, *n* = 12). Dashed line indicates TEER threshold of 50 *Ω* cm^2^ to designate a successful endothelial barrier. **(B)** Permeability of IEC monolayers to 10 kDa dextran when cultured on either 0.4 μm track etched material (COTS) or DL06 electrospun material. Permeability is reported as percent of dextran transferred across membrane with tissue relative to membrane without tissue. Statistical significance was determined by unpaired *t*-test (*n* = 6 DL06; *n* = 3 COTS). **(C)** Representative immunofluorescence image of antibody staining against ZO-1 (red) and GP2 (green) and bound with markers of actin (white) and DNA (blue) of an epithelial monolayer on electrospun DL02 membrane. Scale bar = 25 μm. **(D)** Representative immunofluorescence image of antibody staining against GP2 (green) and SpiB (red) and bound with markers of actin (white) and DNA (blue) of an epithelial monolayer on electrospun DL02 membrane. Scale bars = 25 μm. **(E)** Representative reconstruction of the depth of the epithelial monolayer derived from a Z stack of images from staining as described for D. Scale bar = 10 μm.

Epithelial monolayers grown on the electrospun materials were characterized to understand two key functional aspects: barrier function and cell type differentiation. TEER was maintained greater than 50 Ω*cm^2^ across multiple electrospun material runs and independent experiments (Figure 6A). In our experience, human ileum TEER *in vitro* varies based on device geometry and membrane material, and therefore our success criteria focused on TEER stability over time rather than absolute value. Some material types supported reproducibly higher TEER than others, and we considered successful TEER when maintained over 50 Ω*cm^2^ after differentiation at culture day 4. Barrier function in mature monolayers was further quantified/confirmed at day 7 by evaluation of the passive diffusion of fluorescein-labeled 10 kDa Dextran. The permeability of monolayers grown on the electrospun material did not significantly differ from that of monolayers on the 0.4 μm COTS Transwell®, although the results were more variable (Figure 6B). Formation of a tightly organized monolayer was visualized through immunofluorescent imaging of the ZO-1 tight junction protein (Figure 6C). To support the ability of DCs to sample the luminal side of the barrier, we hypothesized that M cells would be critical, and therefore induced differentiation of M cells in the monolayers via RANK-L supplementation. After 4 days of differentiation, the expression of M cell marker genes GP2 (mature M cells) and SpiB (immature M cells) was apparent and indicated scattered M cell differentiation (Figures 6D,E). As a result, we determined that it is possible to grow intestinal epithelial monolayers on the electrospun material with functional barrier formation and potential fenestration provided by M cells.

**Figure 7:**
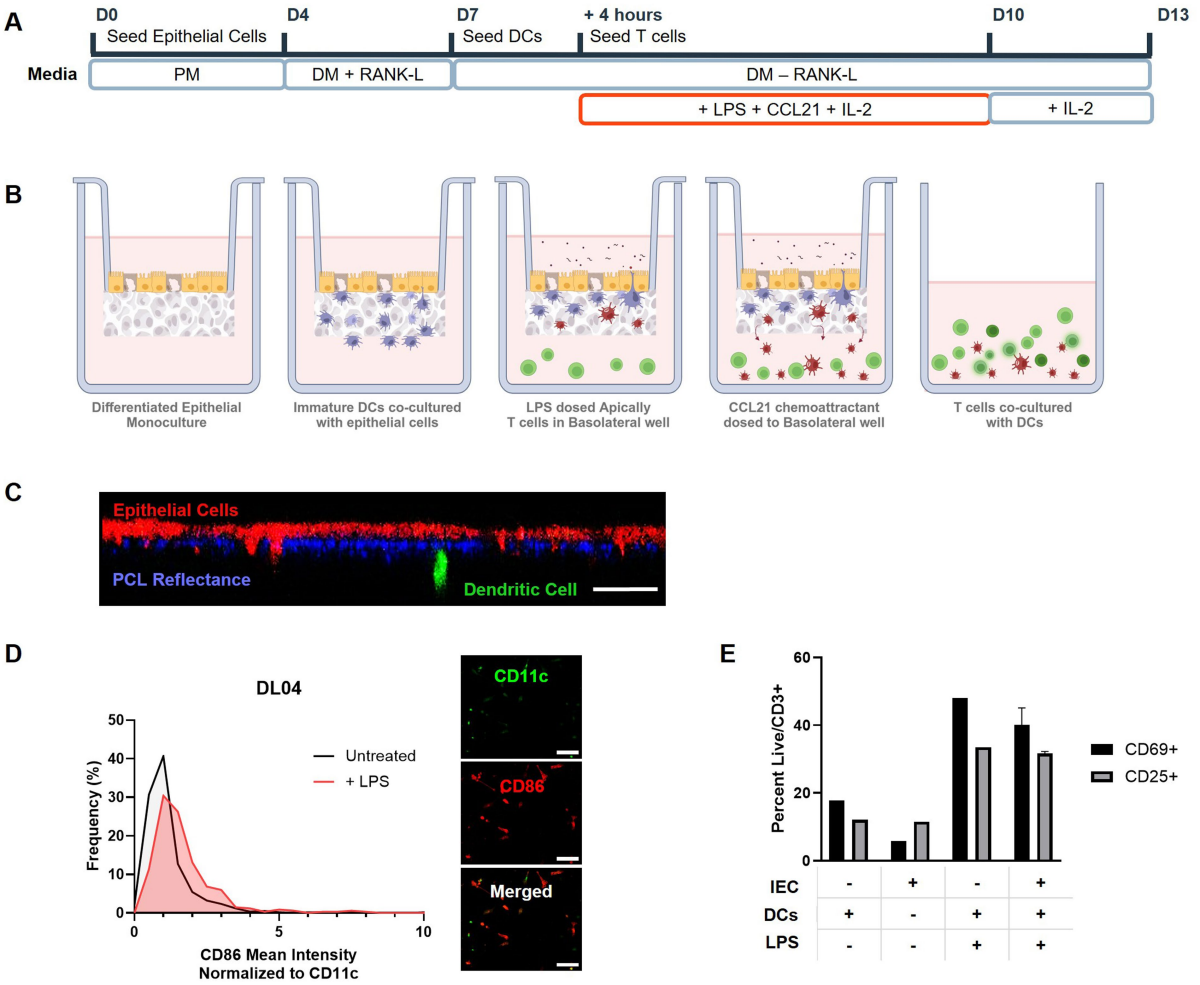
Epithelial-immune cell interactions on the PCL electrospun material. **(A)** Experimental timeline with **(B)** schematic. **(C)** Live cell imaging of immature DCs (green) and IECs (red) 4 h after inverted seeding on PCL material DL01 (blue). Reconstructed orthogonal view of epithelial-immune co-culture imaged at 40X in serial focal planes, scale bar = 25 μm. **(D)** Immunofluorescent images of DCs cultured on PCL material DL04 with IECs and stained with CD11c-FITC and CD86-AF647 on day 8 of cell culture, with or without LPS exposure. Mean fluorescence intensity of CD86 for each cell is normalized by mean fluorescent intensity of CD11c, as determined by ImageJ. The frequency of the normalized CD86 intensity is depicted in a histogram, showing a significant shift (*p* < 0.001) increase for cultures treated with LPS. Statistical significance determined by *t*-test. Representative images are shown of a sample treated with LPS; scale bars represent 100 μm. **(E)** Flow cytometry characterization of T cells after 13 days of culture. Cells were stained for CD3^+^ (T cell general marker), CD69^+^ (T cell activation early stage) and CD25^+^ (T cell activation late stage). Cell culture models to which the T cells were exposed were IECs alone (no DCs), DCs alone (no LPS exposure), DCs alone with direct LPS exposure, and DCs plus IECs with LPS exposure. All culture conditions are *n* = 1 devices except for tri-cell culture (*n* = 2 devices). Created in part with BioRender. Walker, J. (μ25) https://BioRender.com/3n3j10n.

Differentiated monolayers were achieved by day seven of culture, similar to the maturation timeline required for monolayers grown on Transwell® devices. Immune cells were integrated by seeding onto the porous side of the inverted device for 1–4 h, before the devices were re-oriented and fresh media added. LPS was then added as a bacterial mimetic. DCs were recovered from the electrospun material with the chemoattractant CCL21, which enhances DC migration, consistent with its role in the lamina propria (54) (Figure 3). The extent of DC maturation was measured by *in situ* staining or co-culture with naïve T cells (Figures 7A,B). First, we evaluated the potential for DCs to situate proximal to epithelial cells. Prior to LPS induction, pre-stained immature DCs were introduced into the 3D scaffold and imaged 1 h later. Intermittent DCs were observed at the PCL surface-epithelial monolayer interface, demonstrating the potential for direct contact between dendritic and epithelial cells (Figure 7C).

After integration of DCs into the electrospun material, LPS was added to the apical side of the epithelial monolayer. Prior data indicated that similarly sized molecules are restricted from transfer through the mature epithelial tissue (Figure 6B). Therefore, passive exposure of DCs to LPS was unlikely. 24 h after LPS dosing, maturation of the DCs was assessed by immunofluorescent staining for CD11c (all DCs) and CD86 (mature DCs only) and imaging of the porous side of the electrospun material. CD86 expression was higher in the devices that received LPS compared to those that did not, where the quantification is presented as a histogram that indicates a shift towards higher intensity CD86:CD11c staining (Figure 7D). This increase in CD86:CD11c mean intensity is not calculated here but is clearly lower than the three-fold increase observed when DCs monocultured in 2D on glass-bottom culture wells were treated with LPS (not shown). This difference may be the result of challenging conditions for evaluating the DCs closest to the epithelial cells given the optical impenetrability of the PCL compared to the DCs imaged on the 2D glass surface. As a functional evaluation of DC maturation, co-cultured T cell activation was measured with markers for early (CD69) and late (CD25) activation. Both activation markers were increased in cultures with DCs exposed to LPS compared to those not exposed. Critically, the levels of T cell activation were as high in the presence of the epithelial barrier as without, which allows direct exposure of DCs to LPS (Figure 7E). T cells exposed to either unstimulated DCs or the epithelial monolayer did not show evidence of activation. These results indicate that the electrospun material provides a functionally different epithelial:DC co-culture context than simple porous membrane and can support more relevant exposure of the immune system to potential stimulants. This tissue culture system thus provides the opportunity to study the effects of luminal contents on target immune cells in the physiologically relevant context of a healthy epithelial barrier.

## Discussion

We set out to develop a platform that could help characterize the epithelium-controlled exposure of immune cells to intestinal lumen contents. Our motivation was eventually to support studies related to food allergies, microbiome influence on the immune system and oral vaccines. In the first steps towards this goal, we aimed to derive a physiologically relevant material to support both epithelial cells on a two-dimensional surface and immune cells in a three-dimensional context and enhance interaction between the two cell types. Our hypothesis was that achieving these goals would result in a tissue culture system that would maintain the epithelial barrier and support immune cell activation, using pro-inflammatory stimuli as the test case. We believe that this new platform provides a convenient, scalable system to study human intestinal mucosal health, where the 3D material could support integration of additional immune cell types, enteric neurons and fibroblasts.

We chose electrospinning for matrix fabrication due to the ease of generating matrices from biocompatible materials. Prior reports indicated success using electrospun PCL to support DC culture (52). The use of electrospinning allowed iterative development and tuning of properties through multiple parameter changes, including solution viscosity, composition, spin speed, and distance from the collector (55). Electrospinning is readily scalable (typically producing letter-sized mats), with potential to scale further (roll-to-roll processes). It should be noted that there are limitations to the technique, including requirements for experimental control such as humidity (56). Our custom platform augments a recent body of work combining 3D-printed custom cell culture inserts with tissue matrix materials, including hydrogels (57, 58), spider silk (59), and electrospun nanofibrous materials (60).

### Material factors that influence cell culture

Initially, we integrated the epithelial and DCs separately into our electrospun PCL materials and established relevant readouts to identify material features appropriate for each cell type. We were able to achieve several target metrics, including material thickness, surface pore size and bulk material pore size. Early in our studies, we determined that each cell type requires different pore sizes and chose to solve this by using two different PCL concentrations to derive this variation. More subtle changes in pore size could be made by controlling collector rotation speed. We fully characterized eight material types, and this range provides an opportunity to identify features that may particularly influence the successful culture of each cell type. One material product (DL02) was particularly successful at supporting both the epithelial monolayer and incorporating immune cells.

The impact of material stiffness on DC behavior has been documented; hydrogel stiffness influences DC compatibility (61) and stiffer hydrogels increase DC pro-inflammatory activity (62). The electrospun materials all had a typical modulus 50-fold lower than standard track etched membrane, and relative to these off-the-shelf materials, the electrospun material represented a narrow range of modulus. Within this narrow range, DL02, an electrospun material successful for cell culture, had the highest bulk tensile modulus. The electrospun materials were also 1,000-fold stiffer than lamina propria, and we propose that incorporating collagen (52) into the electrospinning process could further soften the material, approaching the natural context of human intestine.

Our analysis of eight electrospun materials across multiple material features allowed us to correlate successful cell culture with these factors to identify important factors. Electrospun mat thickness had some impact on immune cell integration (Figure 5E), with thicker mats correlating with lower success of integration. Due to the opacity of the mats, we were unable to image and gather specific information relevant to DC migration/chemotaxis in the 3D scaffolds. Pore size appeared to be the most significant factor driving cell culture success, specifically the need for larger pores to encourage the introduction of immune cells and smaller pores to support the formation of an epithelial barrier. These factors highlight the benefit of working with a material with thickness that can support distinct environments for each cell type. Electrospun material is one solution to this challenge.

### Enhanced epithelial:immune cell co-culture

Our rationale for undertaking development of a 2D:3D material was in part the recognition that the physical barrier provided by typical track etched membrane separates cells on either side such that they cannot interact in a physiological manner. In the case of the intestinal epithelial and innate immune cells, particularly in the small intestine Peyer’s patches, intimate connection between the two cell types is required to support luminal sampling and responsiveness by the immune system. Other systems that avoid track etched membranes and integrate 2D and 3D cell types include silk mesh and formed hydrogel structures. Silk scaffolds can be seeded with human IECs on the surface (34, 35, 63) and immune cells integrated into the underlying mesh layer (64), providing a useful co-culture platform. However, the geometry and culture method do not support epithelial barrier assessment by TEER, molecular permeability, or compartment-specific media and treatments. Therefore, the experiment design limits compartmentalized investigation and prevents decoupling results between epithelial cell signaling-mediated and direct exposure of antigens to the immune cells. Additionally, the silk scaffold material is limited in imaging depth.

An alternative method that provides potential direct interaction between immune cells and epithelial cells involves seeding immune cells within the extracellular matrix typically used to create the surface on which the epithelial cells are grown. Two examples of this approach have shown that the two cell types can be co-cultured in this context and will respond to each other through relevant signaling methods. One version builds a thick base of collagen suspended on a highly porous mesh (36). Epithelial cells are cultured on the top surface of the collagen, and a bolus of immune cells can be introduced into the bottom surface of the collagen, from where they migrate towards the epithelial cells. To date, this method has been demonstrated with primary human colon epithelial cells paired with neutrophil-like cells derived from the HL-60 cell line, and it remains to be seen how other, primary immune cell types would function in the collagen base. Similarly, immune cells have been positioned in the extracellular matrix layer supporting the epithelial cells in the OrganoPlate platform, where immune cells are seeded at the time of matrix introduction, and epithelial cells are layered into the platform (65). Use of this commercially available, multiplexed platform provides opportunities for increased throughput and consistency but is hindered by the timing of immune cell introduction during matrix deposition. This system has been developed with epithelial and immune cell lines rather than primary cells, and it is possible that additional efforts could increase the relevance and flexibility of this system. While these systems provide sophisticated options for tissue culture, the dual porosity electrospun PCL adds a different material option, which has been demonstrated with primary human epithelial and immune cells (Table 2).

**Table 2:** Some of the attributes of select epithelial:immune co-culture devices.

System	Transwell	Silk scaffold	Suspended hydrogel	Electrospun PCL
Direct contact epithelial:immune cells		X	X	X
Human primary cell culture demonstrated	X	X		X
TEER and permeability evaluation	X		X	X
Decoupled compartments for treatment and secretion studies	X		X	X
Flexible timing of cell addition	X	X	X	X
Commercial availability	X			

*In vitro* models to study immune response to nutrients and other luminal contents require relevant permeability of the barrier tissue, and TEER is a straightforward way to quickly and non-destructively estimate relative permeability. Comparison of absolute TEER among models is not appropriate because the form of each device and the tool for measurement are variables. In addition, measurement of TEER in human ileum tissue is challenging given the tissue location, small size of typical biopsy, low viability *ex vivo,* variable methodologies and typical reporting as relative rather than absolute values (66–68). In cases where comparative studies of human biology have been performed, it is apparent that ileum has the lowest TEER compared to other small intestine and colon segments, with values of 10–100 Ω*cm^2^ for ileum reported (69, 70). Acknowledging the caveats above, we believe that relatively low TEER indicates ileum-relevant high permeability, and that our demonstration of 2.5-10X lower TEER in the electrospun material compared to Transwell®, devices with similar format, size and measuring tool, implies more relevant permeability.

Epithelial-immune cell interactions are supported in this context, through both direct contact and diffusion of secreted molecular signals. Direct evaluation of mature DCs (mDC) relied on staining and imaging maturation markers on mDCs, and increased CD86 staining intensity was measurable, but not compelling. It is unclear whether this result is a byproduct of evaluation of mDCs only at the material bottom surface, farthest from stimulation. As an alternative evaluation, we used recovery of mDCs from the matrix. This required the introduction of a chemoattractant specific to mDCs, and this hindered our ability to evaluate the extent of maturation since only mDCs would be expected to migrate in response to CCL21. However, we were able to show that this maturation was sufficient to drive activation of T cells, providing a phenotypic readout of the functionality of the epithelial-immune signaling. The structure of the platform as a hanging basket in a typical well plate provides the opportunity to address the apical side of the epithelium separately from the basal side, key to understanding the role of the epithelial barrier in protecting and permeating in response to luminal contents such as nutrients, commensal microbe metabolites and toxins. We envision the opportunity to study the microbiome and its relationship to the host immune system using this platform. Finally, the platform and the cell culture methods are scalable; the electrospun material is produced as a sheet that can yield up to 250 individual inserts, and the introduction of both epithelial and immune cells into each unit is consistent and extendable.

We acknowledge that this model is currently limited with only epithelial cells, DCs and T cells integrated. The innate immune response is orchestrated though multiple cell types, which mature in the tissue context and in contact with each other. Lack of mast cells and macrophages may reduce the sensitivity to allergic response. Lack of cell types that populate the lamina propria, such as fibroblasts, may reduce the contextual maturation of introduced cells. Without circulating immune cells, there is not a pool of naïve cells to infiltrate the tissue and indicate amplifying immune reactions. We envision opportunities for multiple enhancements to the methods we have presented. Access to immune cells derived from donors with known specific sensitivities would allow testing with relevant allergens. The introduction of fibroblasts into the mesh could support epithelial maturation and longevity, and the inclusion of monocyte-derived Macrophages in addition to the DCs would create a more relevant innate immune milieu. The introduction of media flow, particularly into the lower chamber through development of a pumping system, would likewise enhance the longevity and maturity of the system and could provide a route for immune cell introduction. The production of mucus by epithelial cells is a key aspect of barrier function and lumen interaction; mucus-producing cells are present in our system, but we have not fully evaluated nor actively stimulated mucus secretion. Finally, variation of the material used for the electrospinning to include additional or alternate material such as polycarbonate or thermoplastic polyurethane could affect both immune cell integration and migration as well as depth of visualization, which would better support direct measurement of DC maturation. While LPS was used in this work as an accessible and reliable immune activator, our long-term goal is to evaluate food-derived molecules, microbial metabolites, toxins and vaccines. These stimuli may be best matched by the integration of additional host cell types, such as Mast cells, adaptive immune cells, enteric neurons and fibroblasts, all of which may benefit from the 3D nature of the electrospun material.

## Conclusion

We have developed a new approach to integrating intestinal epithelial and immune cell types. Our goal was to provide a platform that accurately represents the interaction of innate immune cells with luminal contents and the resulting downstream inflammatory responses. The epithelial barrier provides a critical element of this response, and our ability to integrate this layer, particularly the immune-regulating Peyer’s patch M cells, provides a relevant context. Our work with electrospun materials allowed us to identify pore size as important to integration of DCs with epithelial barriers, specifically that large pores support immune integration while epithelial cells require small pores for successful monolayer development. Electrospinning provides a method that can be modulated to integrate multiple pore sizes across the depth of the material, thereby providing an option for this type of support material. This system is the first step towards a reproducible and relevant testing platform to support studies of nutritional sensitivities, dysbiotic microbiomes and pathogen-derived toxins.

## Data Availability

The raw data supporting the conclusions of this article will be made available by the authors, without undue reservation.
