# Supplementary material for: A multidimensional platform to support intestinal epithelial and immune cell co-culture
Source: Front Nutr. 2026 Jun 24;13:1769338. doi: 10.3389/fnut.2026.1769338 (PMC13344492; doi:10.3389/fnut.2026.1769338)
Supplement: Supplementary file 1 [file Data_Sheet_1.PDF]

Supplemental Figure 1

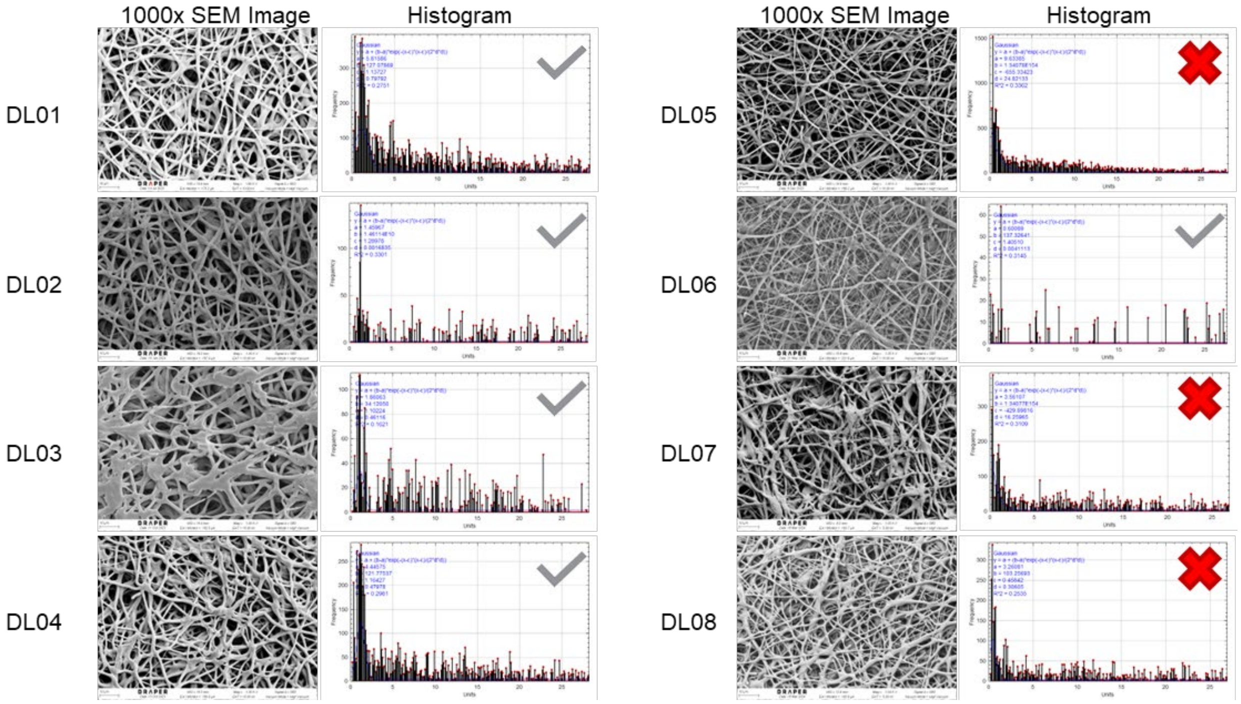

Supplemental Figure 1: The images that were run through the GIFT Image-J Macro for each mat are shown alongside the frequency histogram produced by the data analysis tool (frequency of fiber diameter in microns). Images that were successfully analyzed by the macro are decorated with a green checkmark, while those that failed the analysis because they were reported to have average fiber thicknesses in the range of negative hundreds of microns are decorated with a red x. Qualitatively, the histograms of failed mats tend to have a large, single frequency peak closest to 0, while passing mats have a denser range of large peaks slightly further from the origin (approx. 1-2 um).

Supplemental Figure 2

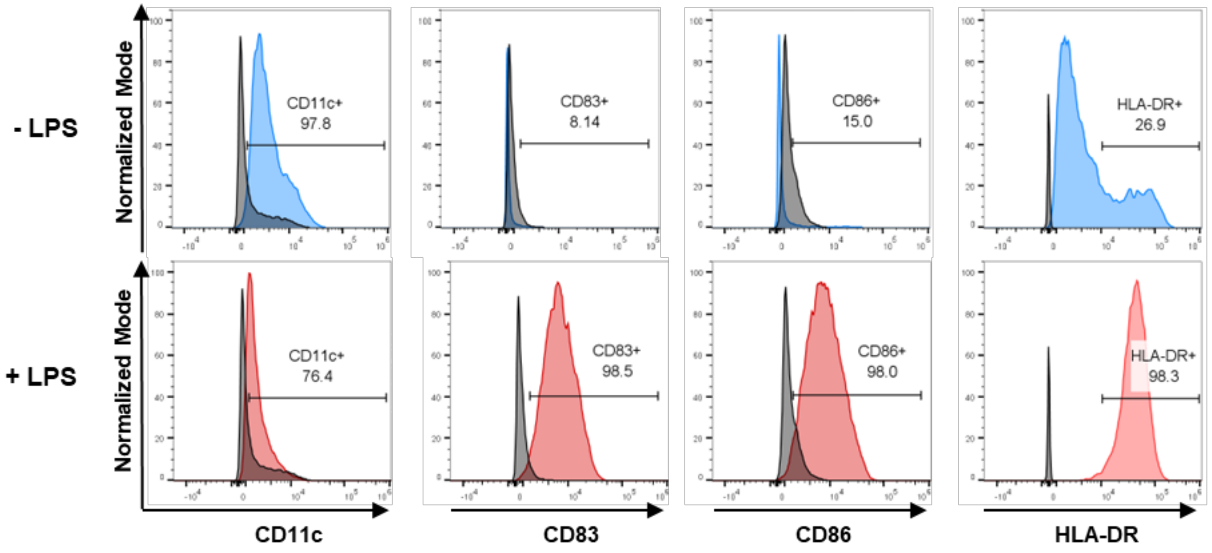

Supplemental Figure 2: Characterization of primary human monocyte-derived DCs by flow cytometry after 6 days of differentiation. DC differentiation was identified by $CD11c^+$ and mature DCs by $CD83^+$, $CD86^+$, or $HLA\text{-}DR^+$. Lipopolysaccharide (LPS) was applied in some cases (blue indicates -LPS culture and red +LPS culture). Histograms in grey indicate Fluorescence-Minus-One (FMO) flow cytometry antibody control for a matched sample.

Supplemental Figure 3

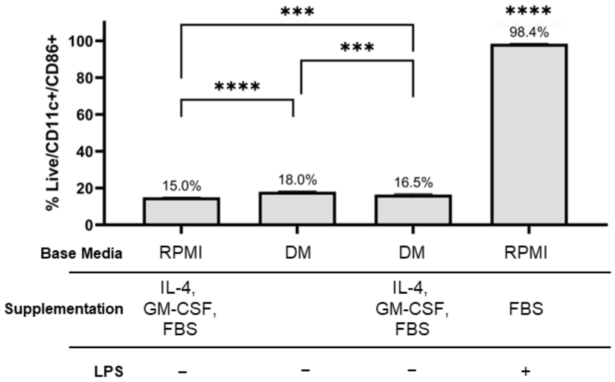

Supplemental Figure 3: Flow cytometry-based characterization of DCs cultured in RPMI or intestinal epithelial DM with additional factor supplementation of IL-4, GM-CSF, and FBS as indicated. LPS was added as indicated to define a window of high activation. The minor, although significant, increase in CD86+ DCs with DM culture still provides a large activation opportunity to the fully activated level. DCs were cultured for 5 days in control media and exchanged to designated base media +/- supplementation for 48 hours. Bars depict averages and standard deviations (n=3 replicates). Statistical analysis was performed using One-Way ANOVA, post-hoc Tukey's HSD. *** = $p < 0.001$, **** = $p < 0.0001$.
